# Carbon Nanotubes Having Haeckelite Defects as Potential Drug Carriers. Molecular Dynamics Simulation

**DOI:** 10.3390/molecules24234281

**Published:** 2019-11-24

**Authors:** Camila Torres, Ignacio Villarroel, Roberto Rozas, Leonor Contreras

**Affiliations:** 1 Departamento de Computación e Informática, Facultad de Ingeniería, Universidad de Santiago de Chile, USACH, Avenida Ecuador 3659, Santiago 9170022, Chile; ctorres@kpitec.com (C.T.); gatrevolution@gmail.com (I.V.); 2 Laboratorio de Química Computacional y Propiedad Intelectual, Departamento de Ciencias del Ambiente, Facultad de Química y Biología, Universidad de Santiago de Chile, USACH, Avenida Libertador Bernardo O’Higgins 3363, Casilla 40, Correo 33, Santiago 9170022, Chile; roberto.rozas@usach.cl

**Keywords:** defective nanotubes, haeckelite defects, nitrogen-doped carbon nanotubes, doxorubicin encapsulation, drug delivery system, binding free energies, non-covalent interactions, *armchair* nanotubes, *zigzag* nanotubes, fully hydrogenated nanotubes

## Abstract

Carbon nanotubes (CNTs) are valuable drug carriers since when properly functionalized they transport drugs and anchor directly to cancerous tumors whose more acidic pH causes the drug release. Herein, we study the so-called *zigzag* and *armchair* CNTs with haeckelite defects to rank their ability to adsorb doxorubicin (DOX) by determining the DOX-CNT binding free energies using the MM/PBSA and MM/GBSA methods implemented in AMBER. Our results reveal stronger DOX-CNT interactions for encapsulation of the drug inside the nanotube compared to its adsorption onto the defective nanotube external surface. *Armchair* CNTs with one and two defects exhibit better results compared with those with four and fifteen defects. Each haeckelite defect consists of a pair of square and octagonal rings. DOX-CNT binding free energies are predicted to be dependent on: (i) nanotube chirality and diameter, (ii) the number of defects, (iii) nitrogen doping and (iv) the position of the encapsulated DOX inside the nanotube. *Armchair* (10,10) nanotubes with two haeckelite defects, doped with nitrogen, exhibit the best drug-nanotube binding free energies compared with *zigzag* and fully hydrogenated nanotubes and, also previously reported ones with bumpy defects. These results contribute to further understanding drug-nanotube interactions and their potential application to the design of new drug delivery systems.

## 1. Introduction

Drug delivery systems are beneficial because they allow one to increase drug bioavailability and reduce their adverse effects. CNTs are valuable drug carriers. When properly functionalized they can be anchored directly to cancerous tumors. Their synthesis and potential use in a wide range of biomedical applications in the treatment of cancer through drugs such as doxorubicin (DOX) together with the analysis of some of the difficulties associated with CNTs’ structures constitute some of the aspects that have been recently reviewed [1]. In the design of drug delivery systems there are at least two important analysis approaches that contribute to improving the results: (i) the drug-carrier interaction that allows the drug to remain associated with the carrier and not be released before the target is reached; in this approach it is relevant to know the carrier’s structural parameters that might favor the indicated interaction and (ii) the release of the drug once it reaches its destination. Among the various drug release control methods of one of the most appropriate is pH control. For example, experimental data shows when DOX is loaded into a single walled carbon nanotube (SWCNT) that has been functionalized with folic acid and polyethylene glycol (PEG), the DOX binds to the nanotube at physiological pH (pH 7.4) and is released only at a lower pH, i.e., lysosomal pH (pH 4.0), which is typical of the tumor environment [2]. These experiments confirmed the findings of Dai et al., who working with PEG functionalized SWCNTs, further demonstrated that, along with the pH, the choice of a suitable SWCNT diameter could also control the DOX release rate from the nanotube [3]. On the other hand, for multiwalled carbon nanotubes (MWCNTs) functionalized with lactic acid and PEG, in addition to pH and temperature, the degree of functionalization allowed the possibility of controlling the amount of DOX released [4]. In vitro results for DOX conjugates on MWCNT functionalized with maleic anhydride-polystyrene revealed a successful release of DOX at pH 5.0 [5]. Therefore an acidic pH (such as is found in the tumor environment) causes the release of the drug, probably by protonation of the amino group producing drug migration to the aqueous phase. In the actual work we will investigate the first approach to determine the effect of the CNT structural parameters on the DOX-CNT binding energies and DOX-CNT interaction, considering neutral species. 

The presence of defects (for example, rings of a different size other than the typical hexagonal rings, or nitrogen doping), modify CNT properties. 

Defects can be generated by rotating a C-C bond around its center or by adding carbon dimers (C_2_ species). As an example of the first type, are the Stone-Wales defects that generate two heptagons surrounded by two pentagons, known as 5-7-7-5 defects [6]. In the case of the addition of carbon dimers to the surface of a nanotube, different types of rings are generated in function of the way in which the addition occurs and the chirality of the nanotube. For example, when the C_2_ addition occurs in an armchair nanotube, in the axial direction of the nanotube, or when the C_2_ addition occurs in a zigzag nanotube but in the transversal direction of the nanotube, two adjacent pentagons are generated surrounded by two heptagons, which are designated as 7-5-5-7 defects and called carbon ad-dimer defects, or C_2_ defects. Interestingly, in the case of an *armchair* (5,5) nanotube the formation of 7-5-5-7 defects occurs with a formation barrier that is calculated to be much lower than that for 5-7-7-5 defects [7], When the addition of several C_2_ moieties is done symmetrically around the entire circumference of the nanotube, a bulge results and the nanotube is called “bumpy”. If the C_2_ units are added along the entire axis of the nanotube, a “zipper” nanotube results, maintaining the metallic character of the nanotube [7].

Haeckelites was the name proposed by Terrones et al. for defects consisting of ordered arrangements of pentagons, hexagons, and heptagons because of their similarity to the radiolaria drawings of Ernst Haeckel [8]. Haeckelite defects with pentagons and octagons are also known, which are predicted as more conductive than their corresponding graphene-based counterparts [9].

Moreover, GaN haeckelite structures that have octagonal and square membered rings have been studied and through calculations of density functional theory it was predicted that these GaN haeckelite nanotubes are semiconductors [10]. The existence of square-octagonal pairs in BN monolayers ground on Cu (111) was also demonstrated [11]. Recently, a new allotropic carbon family with square rings as a key piece has been predicted through quantum chemistry calculations. [12].

In addition to their conductive properties, 5-5-7 haeckelites and haeckelites doped with N can improve catalytic activity for oxygen reduction reactions what has been predicted through theoretical calculations [13]. Furthermore, calculations of density functional theory reveal that *armchair* nanotubes with haeckelite defects physisorb water inside the nanotube [14]. Density functional theory calculations show that a carbon allotrope such as the T-C8, having tetragonal carbon rings, is predicted to be an indirect semiconductor as many of the carbon allotropes, with potential application in the field of nanodevices [15].

CNTs have been recognized as useful drug carriers since, in addition to their valuable properties, it has been shown that under adequate purity conditions, they are not toxic [16]. One important characteristic for obtaining favorable drug-CNT binding free energies is the diameter of the nanotube, as was evidenced through PM6-DH2, a semi-empirical quantum method and the meta-hybrid density functional theory M06-2X in the ONIOM scheme studies of DOX-CNT non-covalent molecular associations. A value of 14 Å was reported as optimal for *armchair* nanotube diameter [17]. Molecular dynamics simulations ratified this diameter value for *armchair* nanotubes and extended it to *zigzag* and *chiral* nanotubes [18]. On the other hand, atomic force microscopy (AFM) imaging and scanning tunneling microscopy (STM) revealed that DOX adsorption is a cooperative process that forms depressions and protrusions along the nanotube [19]. These works, as well as others carried out with molecular dynamics, were made on nanotubes consisting exclusively of hexagonal rings [20,21].

However, as mentioned, nanotubes can contain different types of rings or defects. Because defects change the properties of nanotubes, it is presumed that this may be the cause of some controversial results found in the hydrogen adsorption energy values reported by different authors [22,23,24]. Effectively, molecular dynamics simulations of DOX-CNT complexes reveal that nanotubes with bumpy defects favor DOX-CNT molecular interactions compared with perfect nanotubes, especially for chiral nanotubes [18]. Although defects are known to change the molecular electronic distribution of nanotubes, in the specialty literature there is a lack of systematic knowledge about how different structural parameters can affect the ability of nanotubes with defects to interact and associate with drugs [25]. It is currently unknown how nanotubes with haeckelite defects could form DOX-CNT complexes stable enough to become efficient drug delivery systems and would also be useful to predict their ability to associate with DOX based on their structural parameters.

In this work we explore DOX-CNT interactions for defective CNTs containing haeckelite defects with square and octagonal rings and properly rank defective CNT’s ability for associating to DOX as potential drug delivery media. As known, DOX is an important anticancer agent [26,27,28,29]. 

Our molecular dynamics results revealed that these defective nanotubes show a dependence on the number of defects in the nanotube and the presence and number of nitrogen atoms as nanotube dopants. It is also observed that the initial position and orientation of the drug in the nanotube affects the drug-nanotube interaction. In addition, the effect of considering Restrained Electrostatic Potential (RESP) charges instead of Mulliken charges is studied. The results predict higher drug-nanotube binding free energy values in the case of RESP charges.

For nanotubes that contain one or two haeckelite defects (called Hk1 and Hk2, respectively), drug-nanotube binding free energies are generally better than for nanotubes with a greater number of defects. The drug-nanotube interaction is predicted more favorable when the DOX is in the region of the defect (called DoxD) and not in the region of the regular part of the nanotube (called DoxR). Better interactions are also predicted when the DOX is oriented with its nitrogen pointing towards the center of the nanotube (called position v1) compared to the inverse orientation (called position v2). Binding free energies are favored if the nanotube is doped with nitrogen, especially in cases doped with 4 nitrogen atoms.

The nanotubes with 4-8-8-4 haeckelite defects, that surround completely the nanotube perimeter, called here, Hk, with 10 to 15 defects each, exhibit drug-nanotube interaction energies for encapsulating the drug in their internal cavity, significantly more favorable than drug-nanotube interaction energies for adsorption of the drug on their outer surface as was already found for both perfect nanotubes and nanotubes with 7-5-5-7 bumpy defects [17,18].

## 2. Results

The effect of the chirality, the diameter and the length of nanotube containing haeckelite defects, its nitrogen doping and the initial position and orientation of the DOX in the nanotube on the DOX-CNT non-covalent interaction energies will be analyzed for *armchair* and *zigzag* defective nanotubes, some of them fully hydrogenated. The effect of RESP vs. Mulliken charges for the DOX and the effect of TIP4P vs. TIP3P model for water will also be considered.

### 2.1. Armchair Nanotubes with Hk Defects

**Figure 1:**
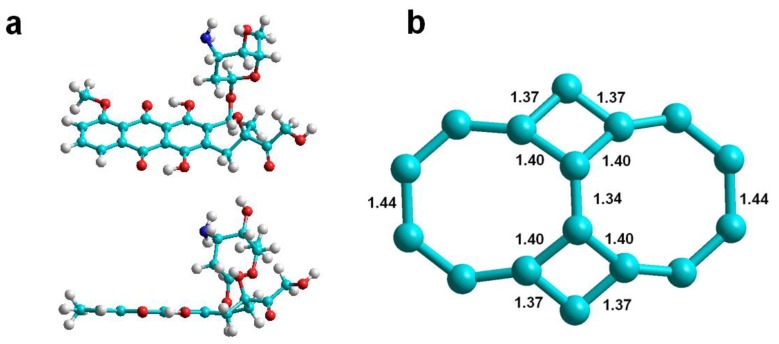
(**a**) DOX structure representation (*C*_27_*H*_29_*NO*_11_**) in frontal and lateral views; (**b**) 4-8-8-4 haeckelite defect representation showing bond distances in Å.

Nanotubes with a maximum number of haeckelite defects, located at the same distance from one of the edges of the nanotube, are designed as Hk nanotubes. Each haeckelite defect consists of a pair of square and octagon rings as shown in Figure 1 where the structure of the DOX is also shown. Herein, A(10,10), A(12,12) and A(15,15) *armchair* Hk nanotubes with approximate diameters of 13, 17 and 21 Å were studied. They had 10, 12 and 15 haeckelite defects each one, respectively. 

**Figure 2:**
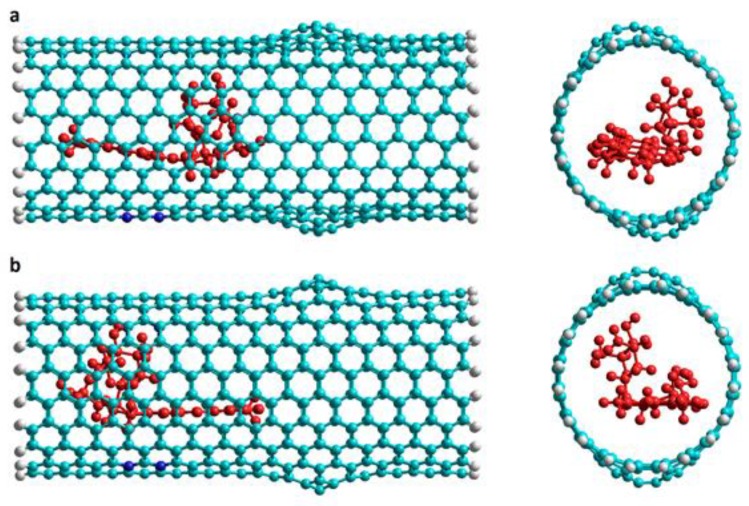
DOX initial orientations when encapsulated in the regular part of an *armchair* Hk2 nanotube. Lateral and frontal views for: (**a**) v1 orientation (NH_2_ group pointing to the center of the nanotube), and (**b**) v2 orientation (inverse orientation). DOX is depicted in red for a better visualization.

Armchair Hk nanotubes exhibit exothermic DOX-CNT binding energies. The more exothermic the DOX-CNT binding energies are, the stronger their interactions will be. *Armchair* Hk nanotubes exhibit stronger interactions for the DOX encapsulation than for the DOX adsorption, as occurs with regular nanotubes (without ad-dimers defects) [17]. However, *armchair* Hk nanotubes with larger diameter favor DOX adsorption with values of PB binding free energies of −42.1 and −50.2 kcal/mol for nanotubes of diameter 17 and 21 Å, respectively (run 7 and 18, Table 1), probably due to the curvature of the nanotube. A smaller curvature facilitates the DOX-CNT π–π stacking interaction for DOX adsorption. Note that the values of PB and GB binding free energies exhibit the same general trend, for that reason only PB binding energy values will be analyzed here. When the DOX is initially located in the region of the defect, it is called DoxD, unlike if it is in the regular region in which it is called DoxR. When the DOX has its nitrogen atom pointing towards the center of the nanotube, its orientation is designated as v1 and if it is in the reverse direction it is designated as v2 as is depicted in Figure 2. 

#### 2.1.1. Nanotube Diameter

The drug-receptor binding free energies for DOX encapsulation depend on the diameter of the nanotube. The most exothermic values of the DOX-CNT binding free energies, which indicate stronger DOX-CNT interactions, correspond to the defective nanotubes of smaller diameter. This is evident when comparing the PB binding free energies obtained for the nanotubes A(10,10), A(12,12) and A(15,15) that have the DOX located initially in the area of the defect and in v2 orientation with values of −79.5, −75.4 and −68.3 kcal/mol, respectively (runs 2, 11 and 20, Table 1). The same trend is observed when comparing the global range of variation of the DOX-CNT binding free energies for the (10,10) smaller diameter systems vs. those of (12,12) intermediate diameter and those of (15,15) greater diameter with PB binding free energy values between −81 and −76 kcal/mol for the smaller CNTs (runs 1–6, Table 1) followed by PB binding free energies with values between −78 and −72 kcal/mol for the intermedium diameter CNTs (runs 7–17, Table 1) and finally by the PB binding free energies with values between −77 and −54 kcal/mol for the largest diameter CNTs suggesting the weakest interactions (runs 18–23, Table 1). 

#### 2.1.2. Nitrogen Doping

**Figure 3:**
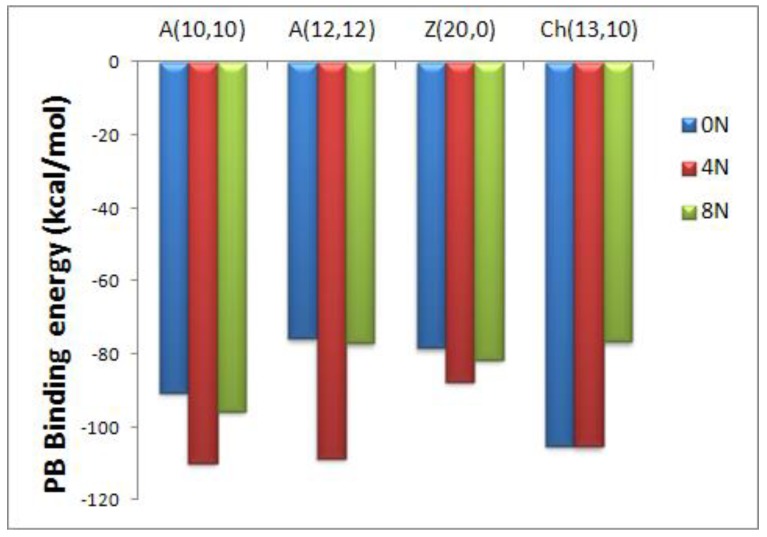
Representation of the Poisson-Boltzmann (PB) binding free energies for encapsulated DOX-CNT complexes of nitrogen-doped and undoped regular *armchair* (10,10), *armchair* (12,12), *zigzag* (20,0) and *chiral* (13,10) nanotubes of diameters 14, 16, 15 and 15 Å, respectively.

The effect of nitrogen doping is revealed as similar for Hk A(10,10) and A(15,15) nanotubes with stronger DOX-CNT interactions for both undoped nanotubes and those doped with 4N that exhibit DOX-CNT PB binding free energy values between −81 and −68 kcal/mol (runs 1–4 and 19–22, Table 1, respectively). Hk nanotubes doped with 8N exhibit the weaker drug-receptor interactions in both cases, A(10,10) and A(15,15), with values of PB binding free energies between −76 and −54 kcal/mol (runs 5, 6, 23, Table 1). The regular A(10,10), A(12,12), Z(20,0) and Ch(13,10) nanotubes showed the same trend predicting stronger DOX-CNT interactions for nanotubes doped with 4N in comparison with those doped with 8N as revealed in Figure 3. For A(12,12) Hk nanotubes, there is no significant trend between nitrogen-doped or undoped nanotubes with values of PB binding free energies between −78 and −72 kcal/mol (runs 11–17, Table 1).

#### 2.1.3. Nanotube Length and DOX Orientation

The effect of the nanotube length depends on the presence of nitrogen. Undoped A(12,12) Hk nanotubes exhibit stronger DOX-CNT interactions when they are longer with PB binding free energies of −75.1 and −72.2 kcal/mol vs. −70.3 kcal/mol for a shorter nanotube (runs 13, 12 and 8, respectively, Table 1). In contrast, A(12,12) nanotubes doped with nitrogen exhibit better DOX-CNT interactions when they are shorter with PB binding free energies of near −79 kcal/mol vs. −78 to −72 kcal/mol for longer nanotubes (runs 9, 10 and 14–17, respectively, Table 1). 

The initial orientation of the DOX in the nanotube does not have a significant preferential effect on the PB binding free energy for the nanotubes with Hk defects. For example for the undoped A(10,10) nanotubes the v1 orientation shows a certain preference over the v2 orientation; for nanotubes doped with 4N, v2 orientation shows a slight preference over v1 orientation and for nanotubes doped with 8N there is practically no preference. 

### 2.2. Armchair Nanotubes with One, Two and Four Haeckelite Defects

**Figure 4:**
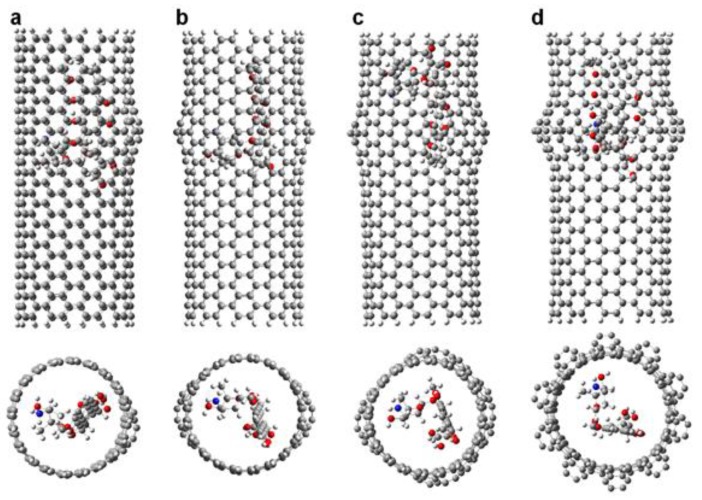
Representation of the DOX-CNT complexes for *armchair* (10,10) nanotubes having different number of haeckelite defects. Lateral and frontal views for: (**a**) one defect (Hk1); (**b**) two defects (Hk2); (**c**) four defects (Hk4); (**d**) the maximum number of defects according to the CNT diameter (Hk), ten defects in the present case.

To analyze how DOX-CNT interactions are affected with the number of defects, we investigated nanotubes A(10,10) with a length of approximately 33 Å having in turn, one haeckelite defect, Hk1, two haeckelite defects, Hk2, located on opposite lateral sides of the nanotube but equidistant from the end, and also nanotubes with four haeckelite defects, Hk4. Figure 4 shows the structures of *armchair* (10,10) nanotubes with 1, 2, 4 and 10 haeckelite defects called Hk1, Hk2, Hk4 and Hk, respectively. 

#### 2.2.1. Armchair Hk1 Nanotubes

The DOX-CNT undoped systems with one haeckelite defect, Hk1, show PB binding free energy values within a range between −92.6 and −90.4 kcal/mol (runs 1–3, Table 2). Nanotubes doped with 4N, exhibit PB binding free energy values of −91.7 kcal/mol (runs 4–6, Table 2) and nanotubes doped with 8N, exhibit PB binding free energy values within a range between −107.1 and −92 kcal/mol (runs 7–10, Table 2). These free energy values suggest stronger DOX-CNT interactions for Hk1 nanotubes in the order: 0N ≤ 4N < 8N, with some preference for 8N-doped systems in which the DOX is initially located in the defect region, with values of the PB binding free energy of −107.1 and −93.3 kcal/mol for v1 and v2 DOX orientations, respectively (runs 7 and 8, Table 2). 

**Figure 5:**
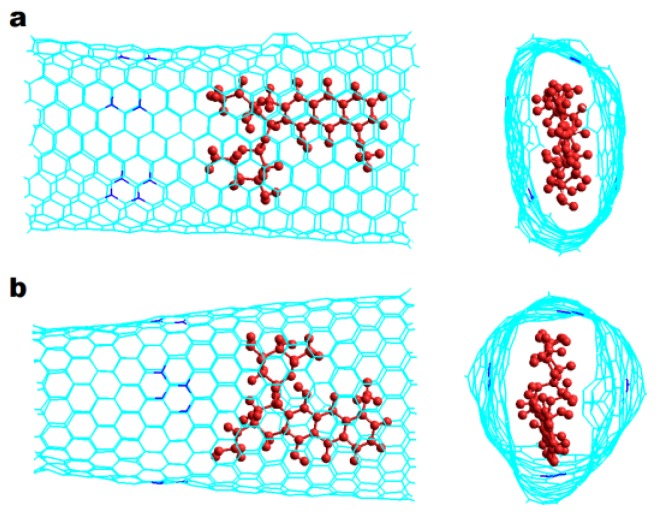
Representation of the DOX-CNT complexes final structures for *armchair* (10,10) and *zigzag* (18,0) Hk1 nanotubes doped with 8N with the DOX encapsulated in the defective part of the nanotube in v1 orientation. Lateral and frontal views for (**a**) A(10,10)-8N-Hk1-DoxD.v1 and (**b**) Z(18,0)-8N-Hk1-DoxD.v1. DOX is depicted in red for a better visualization.

These values also show that the position of the DOX in the nanotube strongly affects the system with 8N favoring the DOX-CNT interaction when the DOX is located in the defect zone with its nitrogen atom oriented towards the center of the nanotube and the flat anthraquinonic system towards the defect zone (v1 orientation, run 7, Table 2). In this case, a greater deformation of the nanotube occurs due to the stronger interaction with the encapsulated DOX, as is depicted in Figure 5a, which translates into smaller d_p-NT_ equilibrium distances (from the plane of the DOX to the nearest nanotube lateral side) and d_N-NT_ equilibrium distances (from the DOX-nitrogen atom to the nearest nanotube lateral side) with values of 3.69 and 3.38 Å, respectively, vs. 3.76 and 3.77 Å for v2 orientation (run 8, Table 2). 

#### 2.2.2. Armchair Hk2 Nanotubes

Undoped nanotubes with two haeckelite defects, Hk2, show values of PB binding free energies ranging between −102 and −91.7 kcal/mol (runs 12–15, Table 2) with stronger DOX-CNT interactions for systems in which the encapsulated DOX is in the regular region of the undoped nanotube (DoxR, runs 14–15, Table 2) and in v2 orientation (run 15, Table 2). Hk2 nanotubes doped with 4N show values of PB binding free energies between −110.2 and −92.4 kcal/mol (runs 16–19, Table 2) and Hk2 nanotubes doped with 8N show values ranging between −94.6 and −92.5 kcal/mol (runs 20–23, Table 2), suggesting that DOX-CNT molecular interactions increase in the order: 8N < 0N < 4N. 

Nitrogen-doped Hk2 nanotubes favor stronger DOX-CNT molecular interactions for systems in which the encapsulated DOX is in the defected region of nanotubes doped with 4N, with values of PB binding free energies of −110.2 and −103.5 kcal/mol for the v1 and v2 orientations, respectively (runs 16 and 17, Table 2). When the encapsulated DOX is in the regular region of nanotubes doped with 4N, PB binding free energy values of −92.4 and −102.8 kcal/mol are obtained (v1 and v2 orientations, respectively, runs 18 and 19, Table 2). Smaller equilibrium distances d_p-NT_ and d_N-NT_ of 3.54 and 3.20 Å, respectively, confirm stronger DOX-CNT interactions for the system with the DOX located in the defected region of nanotubes doped with 4N compared with equilibrium distances d_p-NT_ and d_N-NT_ of 3.88 and 3.71 Å for the encapsulated DOX in the nanotube regular region (runs 16 and 18, Table 2). 

Nanotubes doped with 8N and DOX located in the defected region exhibit PB binding energy values of −92.5 and −94.6 kcal/mol for v1 and v2 orientations, respectively (runs 20 and 21, Table 2). When the DOX is located in the regular region instead, intermediate PB binding energy values of −93.6 and −93.3 kcal/mol for v1 and v2 positions, respectively (runs 22 and 23, Table 2), are obtained. Interestingly, visualization of final snapshot of A(10,10)8N-Hk2-DoxDIn.v2 (run 21, Table 2) using virtual molecular dynamic (VMD) program (http://www.ks.uiuc.edu/Research/vmd/) revealed the same axial orientation of the DOX with a near 50 degree deviation regarding the axial axis of the nanotube as was already observed by means of density functional theory calculations with long–range corrected functional M05-2X for adsorption DOX-CNT complexes of closed *armchair* and *zigzag* nanotubes [19]. 

The results reveal a stronger interaction of the drug with the haeckelite defect zone of the doped CNTs instead of the regular part of the nanotube containing the doping nitrogen atoms. The strongest DOX-CNT molecular interactions, both in the Hk1 and Hk2 nanotubes, are predicted for nitrogen-doped nanotubes when the DOX is in the nanotube haeckelite-defect region and with its nitrogen atom oriented towards the center of the nanotube with PB binding energy values of −107.1 and −110.2 kcal/mol (runs 7 and 16, Table 2) for the Hk1 nanotube doped with 8N and for the Hk2 nanotube doped with 4N, respectively. 

All of the simulations were done considering a TIP3P water model. In order to know if the reactivity ranking of the nanotubes to associate with the DOX was dependent on the water model, some simulations were made using the TIP4P water model that has been shown to provide good results and reproduce solvation free energies [30]. 

**Figure 6:**
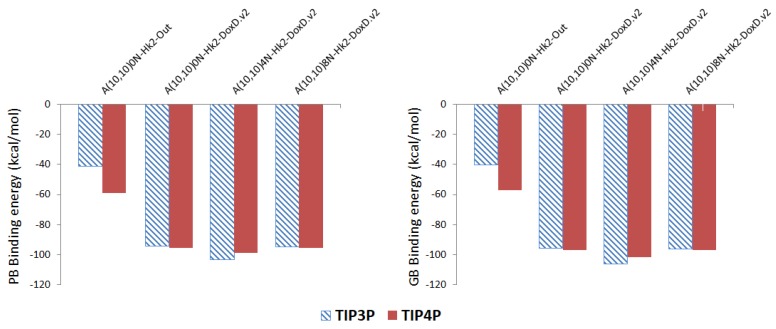
Representation of the Poisson-Boltzmann (PB) and Generalized Bond (GB) binding free energies for DOX-CNT complexes of nitrogen-doped and undoped *armchair* (10,10) nanotubes having two haeckelite defects (Hk2) using TIP3P (hatched surface) and TIP4P (solid surface) models for water.

Figure 6 shows the PB and GB binding free energies of the DOX-CNT complexes, by using the TIP3P and TIP4P water models for the adsorption and the encapsulation of the DOX in *armchair* Hk2 nanotubes, undoped and doped with 4N and 8N. As can be seen, the same trends are found with the two water models TIP3P and TIP4P and for both the PB and GB binding free energy values. This means that the trends found on the nanotubes ranking affinity for DOX adsorption and encapsulation are not dependent on these water models.

#### 2.2.3. Armchair Hk4 Nanotubes 

DOX-CNT binding energies for nanotubes having four haeckelite defects, Hk4, exhibit the same general trend showed for nanotubes with one and two defects predicting more favorable DOX-CNT interactions for nitrogen doped nanotubes with a slight preference for the v1 orientation and with PB values between −88.6 and −82.3 kcal/mol (runs 24–30, Table 2). These energy values are less exothermic than those obtained for nanotubes with one or two haeckelite defects. 

In an attempt to improve the method of building the nanotube models with haeckelite defects and to determine if this factor can affect the results, the unit corresponding to the haeckelite defect was separately constructed and optimized (see Figure 1) and then this optimized unit was integrated into the nanotube in the right place. To the naked eye, a smoothing of the defect was obtained. This feature was signaled using a letter “s” in the nanotube name (Hk4s). Although the smoothing factor studied in the building of defected nanotube revealed a favorable effect in one case, with DOX-CNT PB binding free energies of −82.3 and −87.7 kcal/mol for *armchair* Hk4 and Hk4s nanotubes, respectively (runs 24 and 28, Table 2) for the remaining DOX-CNT binding energies, no significant changes were found. 

The DOX-CNT binding energies depend on the number of haeckelite defects present in the nanotube. Undoped (10,10) *armchair* Hk1, Hk2, Hk4 and Hk nanotubes, with one, two, four and ten haeckelite defects, respectively, exhibit PB binding energy values of −92.6; −94.6; −82.3 and −79.5 kcal/mol. In each of these cases the DOX is encapsulated in the defective part of the nanotube, in v2 orientation (runs 2, 13, 24, Table 2 and run 2, Table 1, respectively). Results reveal that stronger DOX-CNT interactions (more exothermic binding free energy values) are predicted for nanotubes having a lower number of haeckelite defects, being the most favorable the CNTs with two defects. Those systems with less favorable binding free energy values were the Hk nanotubes with a maximum number of defects (10 to 15 defects, according to the nanotube diameter). So, it is possible to rank the defective *armchair* nanotubes following the order: Hk2 > Hk1 > Hk4 > Hk, according to their ability to encapsulate the DOX molecule.

#### 2.2.4. Encapsulation of Multiple DOXs in Armchair Hk1 and Hk2 Nanotubes 

The systems DOX-CNTs that exhibited the most exothermic binding free energies were chosen for testing the effect of increasing the concentration of the encapsulated DOX in nitrogen-doped nanotubes. It was found that a sequential ordering of the DOX molecules in the nanotube doped with 4N, with PB binding energy values per encapsulated molecule of −108.2 kcal/mol (run 3, Table 3), produces more exothermic binding free energies than the system considering the opposite ordering of the DOX molecules in the nanotube, either opposed by the part of the flat anthraquinone rings (DoxOp) with values of −98.6 kcal/mol (run 1, Table 3) or by the part of the nitrogen atoms of the DOX (DoxOpN) with values of −100.6 kcal/mol (run 2, Table 3).

Stronger DOX-CNT interactions for CNTs doped with 4N in relation to those doped with 8N with PB binding free energies per encapsulated DOX molecule of −108.2 vs. −100.6 kcal/mol, respectively (runs 3 and 6, Table 3) are found again. Also, stronger DOX-CNT molecular interactions for the Hk2 vs. the Hk1 nanotubes doped with 8N are predicted with values of PB binding free energies per DOX molecule of −100.6 and −92.8 kcal/mol, respectively (runs 6 and 5, Table 3). 

#### 2.2.5. Saturated Armchair Nanotubes 

As a way of evaluating the importance of the π–π stacking in the DOX-CNT interaction, it was decided to investigate the formation of ligand-receptor complexes using nanotubes unable to form π–π stacking interactions. We evaluated the corresponding fully hydrogenated nanotubes, having sp^3^ hybridization only, against the encapsulation of the DOX for some DOX-CNT systems with one and two haeckelite defects. 

Indeed, the DOX encapsulation results for these saturated nanotubes exhibit significantly less favorable PB binding free energies than corresponding unsaturated nanotubes predicting weaker DOX-CNT molecular interactions. These results constitute an indirect proof of the importance of π–π stacking as a stabilizing force of the DOX-CNT molecular interactions at unsaturated nanotubes. The *armchair* Hk1 and Hk2 saturated CNT doped with 4N are predicted to develop stronger DOX-CNT interactions than the undoped saturated CNT with PB binding free energies of −69.8 and −66.7 kcal/mol (runs 2 and 5, Table 4) compared with −65.1 and −64.3 kcal/mol for the non-doped Hk1 and Hk2 nanotubes, respectively (runs 1 and 4, Table 4). *Armchair* Hk1 saturated CNT, in both cases, are predicted to better encapsulate DOX than corresponding *armchair* Hk2 saturated CNT, as shown in Table 4.

#### 2.2.6. Armchair Complexes Simulations Using RESP Charges for DOX

Restrained Electrostatic Potential (RESP) charges have been used with good results for conformational energy calculations of biological molecules [31]. In this work we investigate how the values of the DOX-CNT binding free energies are affected when using RESP charges for the DOX in order to determine if there are any variations in the ranking of DOX-CNT conjugate stability tendencies established using Mulliken charges. 

Adsorption of the DOX on doped and undoped *armchair* Hk2 nanotubes reveals the formation of weak DOX-CNT molecular interactions with PB binding free energies of −40.5 kcal/mol (runs 7–8, Table 5) when RESP charges are used for DOX. These energies are somewhat less exothermic than the binding free energies obtained when using the Mulliken charges for the DOX with values of −41.1 and −43.2 kcal/mol for the *armchair* Hk2 nanotubes with 0N and 4N, respectively (runs 10 and 11, Table 2). 

Meanwhile, DOX encapsulation in 4N-doped *armchair* Hk1 and Hk2 nanotubes exhibit the best DOX-CNT molecular interactions (using RESP charges for DOX) with values of the PB binding free energies of −83.7 and −98.6 kcal/mol for the Hk1 and Hk2 nanotubes, respectively (runs 2 and 10, Table 5). This behavior found by simulation using the RESP charges for the DOX is practically the same as that found when doing the simulation with the Mulliken charges for the DOX, which validates in a certain way the ranking of reactivity found here for the defective nanotubes with respect to their ability to get associated with the DOX. 

When analyzing the formation of ligand-receptor complexes using saturated nanotubes unable to form π–π stacking interactions, the results, in fact, showed a considerable decrease of the DOX-CNT attraction forces for saturated *armchair* nanotubes with 0N, 4N and 8N. A significant variation of the PB binding free energies towards less exothermic values in the range −59.5 to −61.8 kcal/mol (runs 16–18, Table 5) compared to −81.7 to −83.3 kcal/mol (runs 4–6, Table 5) for unsaturated nanotubes was evidenced. These results contribute to demonstrate the importance of the π–π stacking attraction forces to favor DOX-CNT molecular interactions for nanotubes containing haeckelite defects and in this way to stabilizing the DOX-CNT complexes, in agreement to what was found for nanotubes without defects [17,32]. 

### 2.3. Zigzag Nanotubes with Haeckelite Defects

We studied the ability of *zigzag* nanotubes with one and two haeckelite defects to interact with DOX by means of its external surface (adsorption of DOX) and its internal surface (encapsulation of DOX). All the studied nanotubes had a diameter close to 14 Å, and a length of about 33 Å. The effect caused by the nanotube nitrogen doping on the DOX encapsulation in both the defect region of the nanotube (DoxD) and the regular region (region without defect, DoxR) was studied considering the DOX v1 and v2 orientations described before. Table 6 shows the obtained results analyzed below.

#### 2.3.1. Zigzag Hk1 Nanotubes

The best DOX-CNT interactions occur for the DOX encapsulation in *zigzag* nanotubes having one haeckelite defect (Hk1) in comparison with the DOX adsorption, with values of DOX-CNT PB binding free energies of −39.8 kcal/mol for the adsorption (run 2, Table 6) vs. DOX-CNT PB binding free energy values that fluctuate between −85.5 and −104.6 kcal/mol for the DOX encapsulation (runs 3–8, Table 6). This trend is in agreement with published molecular dynamics simulation results for regular nanotubes and also for nanotubes containing bumpy type defects [18]. Nitrogen-doped *zigzag* Hk1 nanotubes exhibit stronger DOX-CNT interactions than undoped nanotubes, as was the case for *armchair* nanotubes. *Zigzag* nanotubes doped with 4N and 8N exhibit DOX-CNT PB binding free energy values of −108.4 and −106.8 kcal/mol, respectively (runs 7 and 8, Table 6). In Figure 5 final structures for (**a**) *armchair* and (**b**) *zigzag* 8N doped DOX-CNT complexes are depicted. *Zigzag* nanotubes exhibit slightly more stable DOX-CNT complexes than *armchair* nanotubes. In both cases the DOX remains in the defect zone of the nanotube showing a significant nanotube deformation. The corresponding *armchair* Hk1 nanotube doped with 4N exhibits a less exothermic PB binding free energy of −91.7 kcal/mol (run 6, Table 2). 

The DOX-CNT interactions for the undoped *zigzag* Hk1 nanotubes showed dependency on the encapsulation region and on the specific orientation of the DOX, being more favorable those in which the DOX is located in the defect part of the nanotube with its nitrogen oriented towards the center of the nanotube (v1 orientation) with PB binding free energy values of −104.6 and −93.1 kcal/mol for v1 and v2 orientations, respectively (runs 3 and 4, Table 6) and values −93.6 and −85.5 kcal/mol for the same positions in systems where the DOX is in the regular nanotube area (region without any ad-dimer defect; runs 5 and 6, Table 6).

#### 2.3.2. Zigzag Hk2 Nanotubes

The undoped *zigzag* nanotubes with two haeckelite defects, Hk2, exhibit DOX-CNT interactions, unlike the *zigzag* Hk1 nanotubes, and they are favored when the DOX is encapsulated in the v2 orientation either in the defected nanotube region or in the regular nanotube region with values of the PB binding free energies of −99.7 and −94.4 kcal/mol, respectively (runs 15 and 17, Table 6). The corresponding PB binding free energies for the DOX encapsulation with v1 orientation in the defected nanotube region and in the regular nanotube region show values of −85.9 and −92.3 kcal/mol, respectively (runs 14 and 16, Table 6). As seen, the encapsulation of the DOX, either in the regular region or in the defected region, when the DOX is in the v2 orientation (having more exothermic binding free energies), is the one predicted to develop stronger DOX-CNT interactions than when it is in the v1 orientation. 

**Figure 7:**
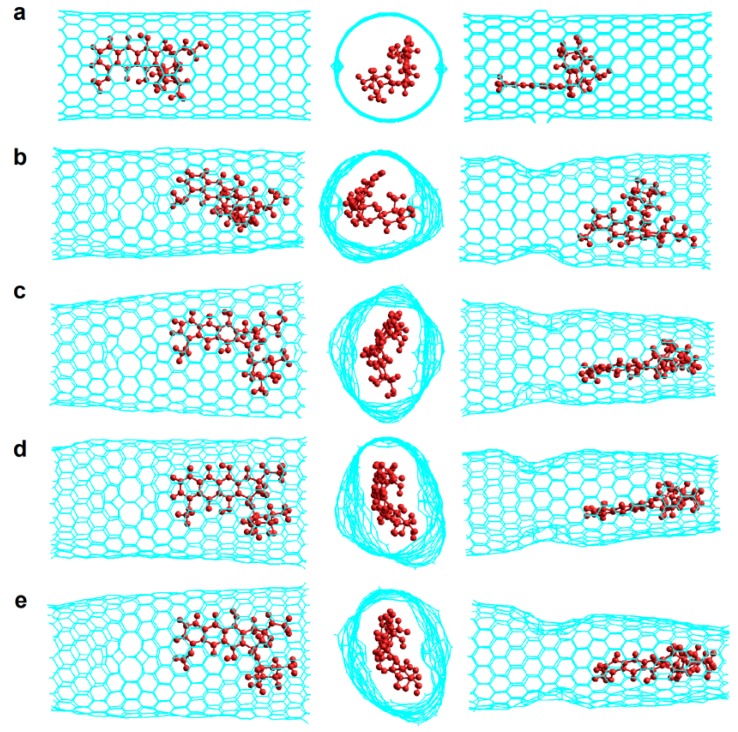
Final structures of Z(18,0)0N-Hk2-DoxDIn.v1 at different simulation times. (**a**) Initial structure (0 ns); (**b**) 2 ns; (**c**) 5 ns; (**d**) 10 ns; (**e**) 15 ns. Left and right are side views; central are front views.

**Figure 8:**
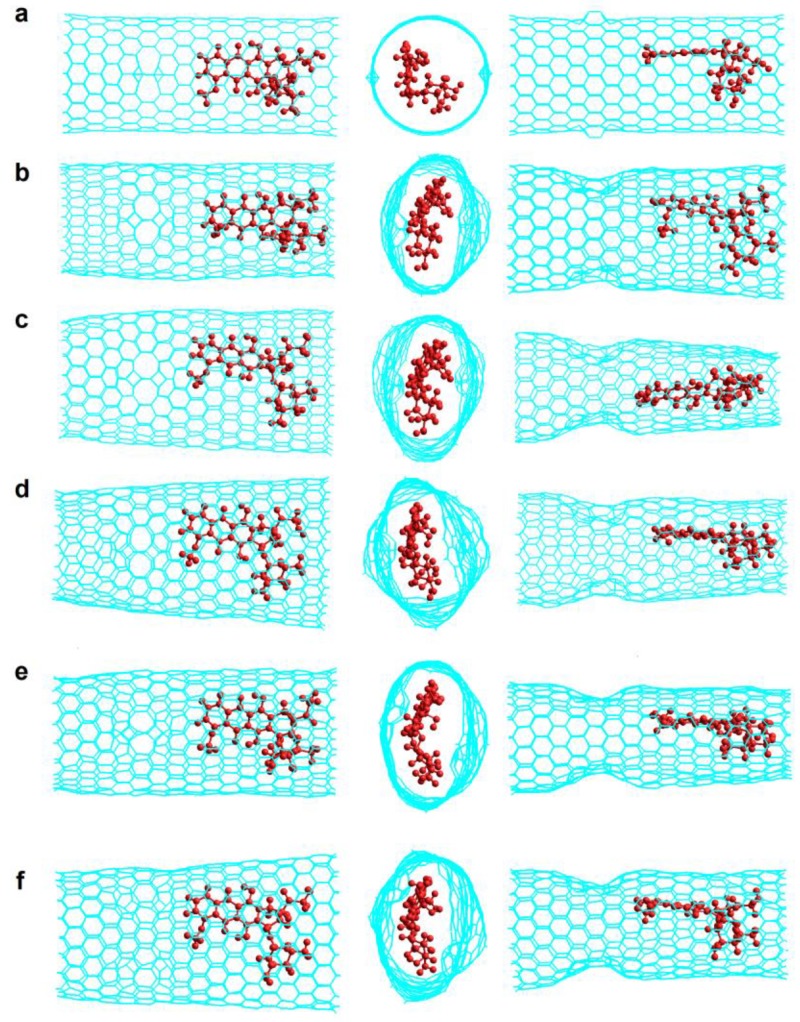
Final structures of Z(18,0)0N-Hk2-DoxRIn.v2 at different simulation times. (**a**) Initial structure (0 ns); (**b**) 2 ns; (**c**) 5 ns; (**d**) 30 ns; (**e**) 50 ns; (**f**) 100 ns. Left and right, are side views; central, are front views.

To analyze in more detail the stability and the molecular interactions of these DOX-CNT complexes, much longer simulations were carried out. Figure 7 shows the final structures of undoped Z(18,0)0N-Hk2-DoxDIn.v1 complex at 0, 2, 5, 10 and 15 ns. It is observed that DOX undergoes a clear change of location moving from the initial region in which the DOX was located in the area of the defects (Figure 7a), to the regular zone of the nanotube (Figure 7b–e). The structures represented on the left and on the right in Figure 7 are the DOX-CNT complex side views. On the left we can see the widest part of the nanotube that shows the four- and eight-membered rings of the haeckelite defects. The views on the right show the nanotube deformation that occurs in the defect region in which the nanotube is clearly deformed. This phenomenon could be partially the cause that the DOX migrates from the area of the defects to the regular zone of the nanotube. The central part of Figure 7 shows a front view and also shows the deformation of the nanotube that adapts to the DOX structure favoring the π–π stacking DOX-CNT intermolecular interactions. The observed DOX displacement to the nanotube regular region occurs in the early stages of the simulation. This behavior is predicted also for the Z(18,0)4N-Hk2-DoxDIn.v1 complex doped with 4N with the DOX moving to the nanotube regular zone as will be shown later. The same happens for the Z(18,0)8N-Hk1-DoxDIn.v1 complex doped with 8N. However, a different behavior is revealed for systems having DOX in v2 orientation where no displacement of the DOX occurred as for instance in the case of Z(18,0)0N-Hk2-DoxDIn.v2 and Z(18,0)0N-Hk2-DoxRIn.v2 complexes (see Figure 8). 

It should be noted that the Z(18,0)0N-Hk2-DoxDIn.v2 complex exhibits the most exothermic PB binding free energy value of the *zigzag* Hk2 nanotubes (−99.7 kcal/mol, run 15, Table 6) and is also the one that presents the smallest equilibrium distance, d_p-NT_, of 3.22 Å (between the DOX hydroxyanthraquinone flat rings and the nanotube wall) which suggests the formation of a DOX-CNT π-π stacking interaction. Additionally, this same complex has one of the smallest equilibrium distance d_N-NT_ of 3.25 Å (between the DOX nitrogen atom and the nanotube wall), suggesting the formation of NH-π hydrogen bond interactions. Also, the systems that exhibit the most exothermic binding free energy values, as shown in Table 6, are those showing the great nanotube deformation, which can be inferred from the distances d_p-NT_ and d’_p-NT_, which together give a value lower than 8 Å considering that the diameter of the initial nanotube model is about 14 Å.

Figure 8 shows the final structures of Z(18,0)0N-Hk2-DoxRIn.v2 complex at different simulation times from 0 to 100 ns. As before, structures represented on the left and on the right are the DOX-CNT complex side views. On the left the widest part of the nanotube showing the particular rings of the haeckelite defects are represented. For example, in Figure 8c at the left, the four- and eight-membered rings are clearly visible, and it is possible to observe also an angle of deviation of about 50 degrees of the DOX tetracyclic flat ring system with respect to the axis of the nanotube. This deviation of the DOX structure was already observed for the adsorption of the DOX on the surface of closed-ended *armchair* (5,5) and *zigzag* (10,0) single-walled carbon nanotubes models by M05-2X/6-31G(d,p) theoretical level studies [19]. 

Table 7 summarizes the corresponding binding free energies and geometric data for Z(18,0)0N-Hk2-DoxRIn.v2 and Z(18,0)0N-Hk2-DoxDIn.v1 complexes at different simulation times. The DOX-CNT PB binding free energy (−99.0 kcal/mol) keeps itself without any variation in the last 70–80 ns as it is shown in Table 7 for the first system. For the second system it can be noted that simulations longer than 5 ns exhibit the same DOX-CNT binding free energy value of −87 kcal/mol. 

Zigzag Hk2 nanotubes doped with 4N (Z(18,0)4N-Hk2-DoxDIn.v1) with a PB binding free energy value of −98.0 kcal/mol (run 18, Table 6) suggest the formation of very strong DOX-CNT interactions. In this case, the same behavior shown by the undoped DOX-CNT complex is observed and the DOX that initially is in the defect zone moves to the regular zone of the nanotube. The dopant nitrogen atoms are found in this zone. The equilibrium distances d_p-NT_ and d_N-NT_ with values of 3.4 Å each, suggest the formation of hydrogen bond attractive forces in addition to the π–π stacking intermolecular attraction forces.

**Figure 9:**
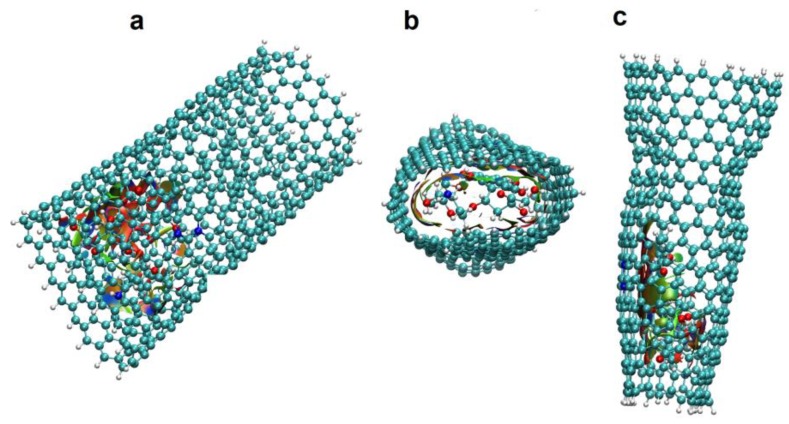
Representation of non-covalent interactions, NCI, for final Z(18,0)4N-Hk2-DoxDIn.v1 complex structure. (**a**,**c**) are lateral views; (**b**) is frontal view. Blue surfaces show DOX-CNT strong interactions; in green, weak interactions and in red, repulsion interactions.

Figure 9 shows the non-covalent interactions (NCI) that take place between the DOX and the nitrogen-doped *zigzag* Hk2 nanotube at the molecular dynamics final structure of the system Z(18,0)4N-Hk2-DoxDIn.v1 (run 18, Table 6). In NCI specialized methodology the blue color surfaces show the strong interactions, the green surfaces the weak interactions (such as hydrogen bonds and π-π stacking) and red surfaces show the repulsions [33].

Along with the displacement of the DOX towards the regular part of the nanotube and the deformation of the nanotube in the part of the defects, it is observed that one of the most extensive green surfaces corresponds to the π-π stacking interaction between the DOX anthraquinonic system and the CNT rings. Although some red regions are also observed, several blue spots stand out, indicating that there are indeed strong attractive DOX-CNT molecular interactions. For example in the front part of Figure 9a, near the nanotube left end, a blue surface associated with a strong interaction of the DOX nitrogen atom with the nanotube surface is clearly seen. In this way, the relative stability ranking of DOX-CNT complexes in terms of non-covalent interactions and the DOX-CNT binding free energy values found reveal as coherent. By analyzing NCI interactions in Figure 9 it can be inferred that the DOX-CNT attraction forces are constituted by several non-covalent interactions of the type π–π stacking, N-H^…^π and C-H^…^ π. 

#### 2.3.3. Zigzag Complexes Simulations Using RESP Charges for DOX

**Figure 10:**
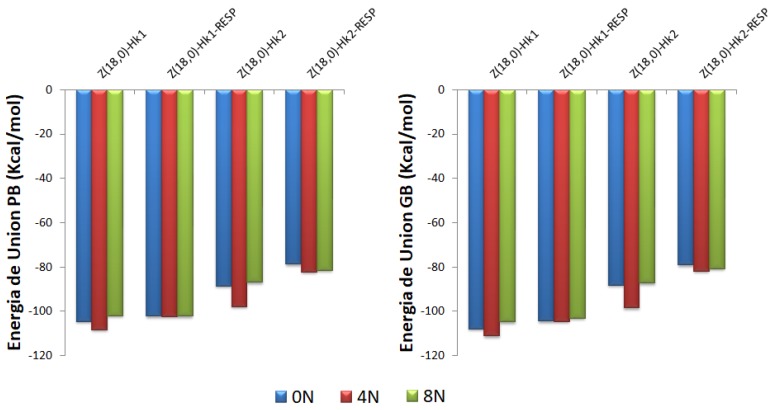
Representation of the Poisson-Boltzmann (PB) and Generalized Bond (GB) binding free energies for DOX-CNT complexes of nitrogen-doped and undoped *zigzag* (18,0) nanotubes having one and two haeckelite defects considering Mulliken and RESP charges for DOX ligand. Each set of data is represented in the order 0N, 4N and 8N, in blue, red and green colors, respectively.

The DOX-CNT binding free energies for the *zigzag* Hk1 and HK2 nanotubes with different nitrogen content obtained in the molecular simulation using RESP charges for DOX have less exothermic values than those obtained when using Mulliken charges and they are depicted in Figure 10, where a comparison of the DOX-CNT PB- and GB binding free energies for the doped and undoped *zigzag* Hk1 and Hk2 nanotubes considering Mulliken and RESP charges for DOX are presented. Although simulation results using RESP charges for DOX tend to attenuate the differences between the nanotube binding free energies (for 0N, 4N and 8N), they show the same tendencies found in systems with Mulliken charges such as: (i) more exothermic values for DOX encapsulation in Hk1 systems compared to Hk2 (for instance, PB binding energy of −102 kcal/mol for Hk1 systems (runs 11–13, Table 6) vs. −78.7 to −82.3 kcal/mol for Hk2 systems (runs 20–22, Table 6); (ii) larger exothermic values (better interactions) for Hk2 systems doped with 4N with PB values of −82.3 kcal/mol (run 21, Table 6) compared with 0N and 8N systems; (iii) significantly more exothermic values for the DOX encapsulation, as seen, compared to DOX adsorption with PB values of −39.7 kcal/mol (run 10, Table 6). 

#### 2.3.4. Zigzag Diamondoid Nanotubes

The main attractive forces that stabilize the non-covalent DOX-CNT interactions correspond to the formation of a π–π stacking interaction that necessarily involves carbon atoms with sp^2^ hybridization of both the nanotubes and the DOX. The evaluation of DOX-CNT binding free energies with saturated nanotubes containing only carbon atoms with sp^3^ hybridization, also called diamondoids, could be an indirect way of verifying the π–π stacking interaction participation. Herein, using the same methodology it was possible to determine that effectively, the non-covalent binding free energies between the DOX (using RESP charges) and the saturated nanotube were significantly less exothermic for the encapsulation of the DOX with values of the PB binding free energies in a range of −55.9 and −63.0 kcal/mol (runs 23–28, Table 6) compared to the values of −78.7 and −82.3 kcal/mol for the corresponding unsaturated nanotubes (runs 20–22, Table 6). 

The orientation of the encapsulated DOX seems to affect the molecular interactions also. It was found that v1 orientation favors DOX-CNT interactions with respect to v2 orientation. For v1 orientation saturated *zigzag* Hk2 nanotubes doped with 4N are the least favorable for DOX encapsulation with a PB binding free energy of −55.9 kcal/mol (run 25, Table 6) in comparison with nanotubes with 0N and 8N. On the other hand, for the v2 orientation, these same saturated *zigzag* Hk2 nanotubes doped with 4N show the most exothermic PB binding energy of −61.1 kcal/mol (run 26, Table 6) when compared with nanotubes with 0N and 8N. 

## 3. Discussion 

The aim of this work was principally to predict a relative affinity scale (or ranking) of the different defective nanotube molecular structures’ binding with DOX. The MM/PBSA and MM/GBSA methods used to carry out the binding free energy calculations overestimate their values because the calculated binding free energies are not the real ones since the unfavorable term of translational entropy has not been considered in their calculation, mainly for reasons of great computational demand. Calculated binding free energies have an average standard deviation of 2–3 kcal/mol. However, the MM/PBSA and MM/GBSA methods revealed as very useful in predicting a ranking of activity against biological systems and have been successfully validated experimentally [34]. These methods have been used to provide a relatively quick estimate of the nanotube activity ranking for DOX encapsulation with consistent results, comparable to other sources and prove to be quite useful if the actual value of the binding free energy is not required [18].

Our results for DOX-CNT binding free energies for nanotubes containing haeckelite defects reveal that those systems with the strongest DOX-CNT interactions are those that exhibit a significant nanotube deformation that favors stabilizing non-covalent interactions, NCI, such as π–π stacking interaction between the DOX anthraquinonic flat part and the nanotube surface integrated with NH–π and CH–π interactions and other van der Waals interactions. This behavior was reported for pure nanotubes and for nanotubes containing bumpy defects also [17,18,32]. Coherently, in most cases, the most exothermic DOX-CNT binding free energy values correspond to systems with the smaller equilibrium distances between the DOX and the nanotube.

It may be difficult to accept that DOX, being a polar molecule, can be stably associated with a hydrophobic molecule such as a nanotube. However, there is experimental evidence supported by analytical techniques of recognized validity (ie, AFM, STM), in different laboratories, which have shown that DOX has ability to load on nanotubes and that their release occurs mainly at acidic pH [2,3,4,5,19]. Although it was surprising at the outset that CNTs allowed high degrees of π-stacking with polar molecules such as DOX [3], examples of π-stacking interactions of CNTs with other polar molecules, such as blood proteins, are known [35].

The non-doped *armchair* (10,10) nanotubes with two haeckelite defects showed the best DOX-CNT binding free energies, with the most exothermic values, between −102.0 and −91.7 kcal/mol. When these *armchair* (10,10) nanotubes systems with haeckelite defects are compared with systems that have bumpy defects (binding free energies of −95.9 to −82.4 kcal/mol) [18], it is found that haeckelite defects confer on the nanotube greater ability to encapsulate the DOX in both D or R positions, in any orientation, v1 or v2. The perfect non-doped *armchair* and *zigzag* nanotubes prefer to be associated with the DOX in v1 orientation, with the amino group of the DOX pointing towards the center of the nanotube. This behavior accounts for the electronic distribution that is established within the nanotube and allows the formation of more stable DOX-CNT molecular interactions. For *armchair* (10,10) nanotubes with haeckelite defects on the contrary, the interactions are especially favored when the DOX is initially located in v2 orientation which was also reported for nanotubes with bumpy defects [18]. The *armchair* (10,10) nanotubes with two haeckelite defects that initially have DOX located in the regular part of the nanotube exhibit the strongest interactions, with PB binding free energies of −95.9 and −102.0 kcal/mol for v1 and v2 orientations, respectively, compared to structures with the DOX initially located near the defect, with PB binding free energies of −91.7 and −94.6 kcal/mol. This behavior was also reported for nanotubes with bumpy defects [18]. This suggests that both types of defects modify the global electronic distribution in a similar way although it is observed that at the end of the simulation, the part of the nanotube where the haeckelite defects are found is modified by moving into the nanotube forming a depression. However, in the case of bumpy defects, the nanotube deforms causing a bulge. This interesting behavior merits further investigation and comparison with other defects. In our research group there is work in progress that considers the study of Stone-Wales defects in the association of nanotubes with DOX. Within the drug delivery system field, the formation of strong drug-receptor attractive forces is considered favorable as a way to prevent the release of the drug from occurring before the nanotube reaches its desired destination or target which obviously favors drug bioavailability and also, reduce the adverse effects in the case that the dose of drug had to be increased to ensure that a proper drug dose reaches the tumors [32].

## 4. Materials and Methods 

Simulations were performed with AMBER16 [36,37] using the combined GAFF and ff99SB force fields. The nanotube models were prepared as single-walled open nanotubes finished in hydrogen with the help of HyperTube [38] and Hyperchem [39]. Perfect nanotubes and those with haeckelite defects with square and orthogonal rings, some doped with nitrogen, were optimized at the Austin Model 1 (AM1) level. The haeckelite 4-8-8-4 defects were built by adding a pair of C ad-dimers in a way parallel to the axial axis of the *zigzag* nanotube. For *armchair* nanotubes instead, ad-dimers were added transversely to the axis of the nanotube. The DOX was optimized at the level of HF/6-31G*. For the specified cases, the restrained electrostatic potential, RESP, methodology was used to derive the partial charges of the DOX molecule determined using the AMBER16 Antechamber program [31,40]. For the rest of the structures the Mulliken charges were used. All the DOX-CNT complexes were neutral systems (as being at the physiological pH of 7.4), solvated in an explicit solvent, in a truncated octahedron water box having 10 Å of spacing distance around the molecule as fixed by the program Leap (Ambertools), using bondi radii. TIP3P was used as the water model. Some simulations, for specific cases, were run using the TIP4P water model. Input files for periodic simulations were prepared with *sander* application programmer interface using dielectric constant default values of 1.0 for all electrostatic interactions, with no restrictions; pH control was not included. Note that in the case of using a continuum electrostatic approach for the calculation of solvation-free energy, both an internal dielectric constant (for the complex) should be used that must be in accordance with the dielectric constant of the MM calculation part; in this case, with a value of 1.0, and an external dielectric constant (for the solvent, water) having a value of 80.0.

After the normal steps of minimization (1000 stages at constant volume), heating (from 0 to 300 K at constant volume), density balance (50 ps at constant pressure) and equilibrium (500 ps at constant pressure), the production step consisting of six independent short stages of 250 ps each at constant pressure was carried out. This procedure was recognized as being efficient and accurate [18,41]. In effect, a 100 ns simulation using independent stages of 5 ns each, revealed similar energy and geometry results as the simulation procedure with short stages described before (see Figure 8). 

The drug (ligand)-nanotube (receptor) binding free energies were determined through the complementary methods MM/PBSA and MM/GBSA implemented in AMBER [36]. In spite that MM/PBSA typically gives too large energies it is recognized as useful in rationalizing experimental and theoretical findings [18,34,41]. These methods were applied on an ensemble of 200 uncorrelated snapshots collected from the equilibrated molecular dynamics simulation and were performed considering $\Delta G={\langle G_{DOX-CNT}-G_{CNT}-G_{DOX}\rangle }_{DOX-CNT}$ where G_DOX-CNT_, G_CNT_, and G_DOX_ correspond to the Gibbs free energy terms for the DOX-CNT complex, the receptor and the ligand, respectively. Each of these terms were calculated considering a number of different terms according to $G=E_{bond}+E_{el}+E_{vdW}+E_{pol}+E_{np}-TS$ wherein *E_bond_* (bond, angle, dihedral), *E_el_* (electrostatic), *E_vdW_* (van der Waals) were the standard molecular mechanics (MM) energy terms, *E_pol_* (polar term) was calculated by solving the Poisson-Boltzmann (PB) and/or generalized Born (GB) equation, *E_np_* (non-polar term) was estimated from a linear relation with the solvent accessible surface area (SA), *T* was the absolute temperature and *S* was the entropy term estimated through normal-mode analysis of the vibrational frequencies, in spite that the normal-mode entropy is expensive to calculate and does not provide information of the conformational entropy change ignoring structural changes in the receptor and ligand upon binding which are supposed to be negligible (one trajectory scheme). 

An exothermic value of the drug-nanotube binding free energy indicates a favorable interaction and the more exothermic the value of the binding free energy will be interpreted as a stronger drug-nanotube interaction.

## 5. Conclusions

We have evaluated the effect of different structural characteristics of *armchair* and *zigzag* single walled carbon nanotubes having 4-8-8-4 haeckelite defects, on their ability to adsorb and encapsulate DOX by means of molecular dynamics simulations with explicit solvent. We found that the DOX-CNT binding free energies depend on nanotube chirality, nanotube diameter/curvature, the number of defects, the nanotube nitrogen doping and the position and orientation of the DOX in the nanotube.

The studied defective nanotubes behave like perfect nanotubes showing the same general trends. In addition, as expected, saturated *armchair* Hk1 and Hk2 nanotubes and saturated *zigzag* Hk2 nanotubes which cannot develop attractive π–π stacking interaction forces, exhibit much weaker DOX-CNT interactions than the corresponding unsaturated *armchair* and *zigzag* nanotubes.

The simulation results allowed to establish the following interesting main trends:

- DOX encapsulation in *armchair* Hk nanotubes is favored for smaller diameter nanotubes meanwhile the DOX adsorption is favored for larger diameter nanotubes, with DOX-CNT binding free energies significantly more exothermic for the DOX encapsulation than for the DOX adsorption.
- *Armchair* nanotubes with a smaller number of defects favor the formation of stronger DOX-CNT interactions. A ranking of their ability to encapsulate the DOX molecule follows the order: Hk2 > Hk1 > Hk4 > Hk.
- Undoped *armchair* Hk2 nanotubes encapsulate the DOX with stronger DOX-CNT interactions than the *zigzag* Hk2 nanotubes with better values for the DOX located in the regular zone of the nanotube, in v2 orientation.
- The best DOX-CNT binding free energy values were found for *armchair* Hk2 nanotubes doped with 4N. The DOX was encapsulated in the nanotube defect region in both the v1 and v2 orientations.

These trends are not altered, in all studied cases, irrespective of whether the simulations are made using: (i) MM/PBSA or MM/GBSA methods; (ii) Mulliken charges or RESP charges for DOX; (iii) TIP3P or TIP4P model for water. This work provides further knowledge regarding the drug-nanotube molecular interactions and the effect of the molecular structure of nanotubes with haeckelite defects on their ability to associate with DOX, knowledge that can contribute to the design of new drug delivery systems based on carbon nanotubes. 

## Figures and Tables

**Table 1:** Poisson-Boltzmann (PB) and Generalized Bond (GB) binding free energy values for DOX-CNT complexes of *armchair* (10,10), (12,12) and (15,15) nanotubes having 10, 12 and 15 haeckelite defects, respectively, and 28 carbon-layers, expressed in kcal/mol. D1 and D2 are the initial CNT diameter at the regular region and at the defected one, respectively, d_p-NT_ is the equilibrium distance between the DOX planar fragment and the CNT sidewall surface, d’_p-NT_ is the equilibrium distance between the same point of the DOX planar fragment and the opposite CNT sidewall surface, and d_N-NT_ is the equilibrium distance between the DOX-nitrogen atom and the CNT sidewall surface. All distances are expressed in Å.

Run	Type	PB	GB	D1	D2	Length	d_p-NT_	d_N-NT_
1	A(10,10)0N-Hk-DoxDIn.v1	−81.3	−80.7	13.3	14.7	32.5	4.26	3.04
2	A(10,10)0N-Hk-DoxDIn.v2	−79.5	−79.6	13.3	14.7	32.5	4.24	3.15
3	A(10,10)4N-Hk-DoxDIn.v1	−78.7	−79.4	13.3	14.7	32.5	3.60	3.58
4	A(10,10)4N-Hk-DoxDIn.v2	−79.4	−79.8	13.3	14.7	32.5	3.30	3.71
5	A(10,10)8N-Hk-DoxDIn.v1	−76.4	−76.4	13.3	14.7	32.5	3.48	3.03
6	A(10,10)8N-Hk-DoxDIn.v2	−76.2	−77.4	13.3	14.7	32.5	3.44	3.45
7	A(12,12)0N-Hk-DoxOut	−42.1	−41.8	16.7	21.8	20.5	3.38	3.77
8	A(12,12)0N-Hk-DoxIn	−70.3	−69.9	16.7	21.8	20.5	3.59	4.37
9	A(12,12)4N-Hk-DoxIn	−78.9	−79.5	16.7	21.8	20.5	3.90	3.28
10	A(12,12)8N-Hk-DoxIn	−79.4	−79.7	16.7	21.8	20.5	3.57	3.64
11	A(12,12)0N-Hk-DoxDIn.v2	−75.4	−74.4	16.8	21.8	33.1	3.67	3.64
12	A(12,12)0N-Hk-DoxRIn.v1	−72.2	−71.4	16.9	21.8	33.0	3.79	3.15
13	A(12,12)0N-Hk-DoxRIn.v2	−75.1	−74.0	16.9	21.8	33.0	3.54	3.22
14	A(12,12)4N-Hk-DoxRIn.v1	−73.7	−72.8	16.7	21.8	33.1	3.76	3.50
15	A(12,12)4N-Hk-DoxRIn.v2	−72.3	−72.0	16.7	21.8	33.1	3.73	4.47
16	A(12,12)8N-Hk-DoxRIn.v1	−78.1	−77.6	16.7	21.8	33.1	3.55	3.04
17	A(12,12)8N-Hk-DoxRIn.v2	−71.6	−70.9	16.7	21.8	33.1	4.04	3.33
18	A(15,15)0N-Hk-DoxOut	−50.2	−49.3	21.1	25.0	33.6	4.18	3.49
19	A(15,15)0N-Hk-DoxDIn.v1	−76.6	−78.3	21.1	25.0	33.6	3.76	3.17
20	A(15,15)0N-Hk-DoxDIn.v2	−68.3	−69.3	21.1	25.0	33.6	3.76	4.17
21	A(15,15)4N-Hk-DoxDIn.v1	−69.9	−68.7	21.1	25.0	33.6	3.48	3.34
22	A(15,15)4N-Hk-DoxDIn.v2	−73.9	−73.6	21.1	25.0	33.6	4.38	3.69
23	A(15,15)8N-Hk-DoxDIn.v2	−53.8	−51.5	21.1	25.0	33.6	5.87	3.45

**Table 2:** Poisson-Boltzmann (PB) and Generalized Bond (GB) binding free energy values for DOX-CNT complexes of *armchair* (10,10) nanotubes having one, two and four haeckelite defects (Hk1, Hk2, Hk4, respectively, with ~33 Å length) expressed in kcal/mol. D1 and D2 are the initial CNT diameter at the regular region and at the defected one, respectively, d_p-NT_ is the equilibrium distance between the DOX planar fragment and the CNT sidewall surface, d′_p-NT_ is the equilibrium distance between the same point of the DOX planar fragment and the opposite CNT sidewall surface, and d_N-NT_ is the equilibrium distance between the DOX-nitrogen atom and the CNT sidewall surface. All distances are expressed in Å.

Run	Type	PB	GB	D1	D2	d_p-NT_	d_N-NT_
1	A(10,10)0N-Hk1-DoxDIn.v1	−91.3	−91.6	13.3	14.6	3.89	3.33
2	A(10,10)0N-Hk1-DoxDIn.v2	−92.6	−93.5	13.3	14.6	3.72	3.73
3	A(10,10)0N-Hk1-DoxRIn.v1	−90.4	−91.5	13.3	14.6	3.59	3.34
4	A(10,10)4N-Hk1-DoxDIn.v1	−91.7	−92.7	13.3	14.6	3.49	3.13
5	A(10,10)4N-Hk1-DoxDIn.v2	−91.6	−94.0	13.3	14.6	3.79	3.13
6	A(10,10)4N-Hk1-DoxRIn.v1	−91.7	−93.3	13.3	14.6	3.62	3.90
7	A(10,10)8N-Hk1-DoxDIn.v1	−107.1	−111.6	13.3	14.6	3.69	3.38
8	A(10,10)8N-Hk1-DoxDIn.v2	−93.3	−94.2	13.3	14.6	3.76	3.77
9	A(10,10)8N-Hk1-DoxRIn.v1	−92.0	−93.1	13.3	14.6	3.76	3.57
10	A(10,10)0N-Hk2-DoxOut	−41.1	−40.3	13.1	15.9	3.39	5.28
11	A(10,10)4N-Hk2-DoxOut	−43.2	−42.5	13.1	15.9	3.43	3.22
12	A(10,10)0N-Hk2-DoxDIn.v1	−91.7	−95.1	13.3	15.5	3.50	4.19
13	A(10,10)0N-Hk2-DoxDIn.v2	−94.6	−95.9	13.3	15.6	4.02	3.81
14	A(10,10)0N-Hk2-DoxRIn.v1	−95.9	−97.8	13.3	16.0	3.53	3.61
15	A(10,10)0N-Hk2-DoxRIn.v2	−102.0	−103.4	13.3	16.0	3.59	2.93
16	A(10,10)4N-Hk2-DoxDIn.v1	−110.2	−115.4	13.3	15.9	3.54	3.20
17	A(10,10)4N-Hk2-DoxDIn.v2	−103.5	−106.3	13.3	15.9	3.68	3.68
18	A(10,10)4N-Hk2-DoxRIn.v1	−92.4	−94.8	13.3	15.9	3.88	3.71
19	A(10,10)4N-Hk2-DoxRIn.v2	−102.8	−104.4	13.3	15.9	3.63	3.43
20	A(10,10)8N-Hk2-DoxDIn.v1	−92.5	−94.2	13.3	15.6	4.08	3.53
21	A(10,10)8N-Hk2-DoxDIn.v2	−94.6	−96.1	13.3	15.6	3.85	3.13
22	A(10,10)8N-Hk2-DoxRIn.v1	−93.6	−96.9	13.3	15.5	3.62	4.05
23	A(10,10)8N-Hk2-DoxRIn.v2	−93.3	−95.1	13.3	15.9	3.64	3.55
24	A(10,10)0N-Hk4-DoxDIn.v2	−82.3	−83.3	14.0	15.5	3.61	3.23
25	A(10,10)4N-Hk4-DoxDIn.v2	−87.5	−90.9	14.1	15.9	4.10	3.03
26	A(10,10)8N-Hk4-DoxDIn.v2	−82.8	−84.5	14.1	15.8	4.48	3.61
27	A(10,10)0N-Hk4s-DoxDIn.v1	−88.1	−88.8	14.0	15.5	3.97	3.71
28	A(10,10)0N-Hk4s-DoxDIn.v2	−87.7	−87.8	14.0	15.5	3.45	2.93
29	A(10,10)4N-Hk4s-DoxDIn.v1	−88.6	−88.8	14.1	15.9	3.90	3.49
30	A(10,10)4N-Hk4s-DoxDIn.v2	−85.9	−87.1	14.1	15.9	4.85	3.44

**Table 3:** Poisson-Boltzmann (PB) and Generalized Bond (GB) binding free energy values for DOX-CNT complexes of doped *armchair* (10,10) nanotubes having one and two haeckelite defects (Hk1 and Hk2, respectively) encapsulating two or three DOX molecules. PB/m and GB/m are the corresponding binding free energies per DOX molecule, all of them expressed in kcal/mol. d_p-NT_ and d′_p-NT_ are the equilibrium distances between the planar fragment of the first and second DOX molecules and the CNT sidewall surface respectively, and d_N-NT_ and d’_N-NT_ are the equilibrium distances between the DOX-nitrogen atom of the corresponding first and second DOX molecules and the CNT sidewall surface. All distances are expressed in Å.

Run	Type	PB	GB	PB/m	GB/m	d_p-NT_	d_N-NT_	d′_p-NT_	d′_N-NT_
1	A(10,10)4N-Hk2-2DoxOp	−197.1	−207.2	−98.6	−103.6	4.04	3.16	4.26	3.71
2	A(10,10)4N-Hk2-2DoxOpN	−201.2	−210.5	−100.6	−105.3	3.66	3.63	3.56	3.56
3	A(10,10)4N-Hk2-2DoxSec	−216.3	−229.0	-108.2	−114.5	3.12	3.43	4.14	3.87
4	A(10,10)4N-Hk2-3DoxIn	−255.3	−270.3	−85.1	−90.1	3.20	3.77	3.09	3.53
5	A(10,10)8N-Hk1-2DoxSec	−185.6	−190.9	−92.8	−95.5	3.54	3.46	3.25	3.65
6	A(10,10)8N-Hk2-2DoxSec	−201.2	−211.2	−100.6	−105.6	3.52	4.73	3.17	3.68

**Table 4:** Poisson-Boltzmann (PB) and Generalized Bond (GB) binding free energy values for DOX-CNT complexes of saturated *armchair* (10,10) nanotubes having one and two haeckelite defects and ~33 Å length, expressed in kcal/mol. D1 and D2 are the initial CNT diameter at the regular region and at the defected one, respectively, d_p-NT_ is the equilibrium distance between the encapsulated DOX planar fragment and the CNT sidewall surface and d_N-NT_ is the equilibrium distance between the DOX-nitrogen atom and the CNT sidewall surface. The DOX was located in the defect zone in v2 orientation. All distances are expressed in Å.

Run	Type	PB	GB	D1	D2	d_p-NT_	d _N-NT_
1	A(10,10)0N-H-Hk1	−65.1	−64.8	15.3	16.1	3.71	3.73
2	A(10,10)4N-H-Hk1	−69.8	−70.0	15.3	16.1	3.95	6.55
3	A(10,10)8N-H-Hk1	−68.4	−69.1	15.3	16.1	3.72	3.27
4	A(10,10)0N-H-Hk2	−64.3	−63.9	15.6	17.7	3.24	3.65
5	A(10,10)4N-H-Hk2	−66.7	−66.1	15.6	17.7	3.92	3.62
6	A(10,10)8N-H-Hk2	−58.0	−60.9	15.6	17.7	4.14	4.53

**Table 5:** Poisson-Boltzmann (PB) and Generalized Bond (GB) binding free energy values for DOX-CNT complexes of saturated and unsaturated *armchair* (10,10) nanotubes having one and two haeckelite defects (Hk1, Hk2, respectively) expressed in kcal/mol. DOX charges were RESP charges. D is the initial CNT diameter at the defected region, d_p-NT_ is the equilibrium distance between the DOX planar fragment and the CNT sidewall surface and d_N-NT_ is the equilibrium distance between the DOX-nitrogen atom and the CNT sidewall surface. All distances are expressed in Å.

Run	Type	PB	GB	D	d_p-NT_	d _N-NT_
1	A(10,10)0N-Hk1-DoxDin.v1RESP	−83.5	−83.4	14.6	3.63	3.27
2	A(10,10)4N-Hk1-DoxDin.v1RESP	−83.7	−84.8	14.6	3.77	5.11
3	A(10,10)8N-Hk1-DoxDin.v1RESP	−82.5	−83.1	14.6	3.83	3.43
4	A(10,10)0N-Hk1-DoxDin.v2RESP	−81.7	−82.9	14.6	4.09	4.29
5	A(10,10)4N-Hk1-DoxDin.v2RESP	−82.6	−84.3	14.6	3.50	3.28
6	A(10,10)8N-Hk1-DoxDin.v2RESP	−83.3	−85.1	14.6	3.76	4.68
7	A(10,10)0N-Hk2-DoxOutRESP	−40.5	−38.7	15.6	3.45	3.55
8	A(10,10)4N-Hk2-DoxOutRESP	−40.4	−38.6	15.9	3.40	4.22
9	A(10,10)0N-Hk2-DoxDin.v1RESP	−83.4	−84.4	15.6	3.35	3.39
10	A(10,10)4N-Hk2-DoxDin.v1RESP	−98.6	−101.8	15.9	3.40	3.74
11	A(10,10)8N-Hk2-DoxDin.v1RESP	−83.9	−85.8	15.6	3.61	3.18
12	A(10,10)0N-Hk2-DoxDin.v2RESP	−84.6	−86.2	15.6	3.81	3.18
13	A(10,10)4N-Hk2-DoxDin.v2RESP	−84.6	−87.1	15.9	3.82	3.46
14	A(10,10)8N-Hk2-DoxDin.v2RESP	−84.5	−86.1	15.6	3.87	3.19
15	A(10,10)0N-H-DoxIn.v2RESP	−62.9	−61.1	32.7	3.64	3.59
16	A(10,10)0N-H-Hk1-DoxDin.v2RESP	−61.8	−59.3	32.7	3.63	3.90
17	A(10,10)4N-H-Hk1-DoxDin.v2RESP	−59.5	−55.3	32.7	3.92	6.49
18	A(10,10)8N-H-Hk1-DoxDin.v2RESP	−60.9	−55.4	32.7	3.21	3.42

**Table 6:** Poisson-Boltzmann (PB) and Generalized Bond (GB) binding free energy values for adsorption and encapsulation DOX-CNT complexes of saturated and unsaturated *zigzag* (18,0) nanotubes with one and two haeckelite defects, considering RESP and Mulliken charges for DOX in v1 and v2 orientations, expressed in kcal/mol. Nanotube diameter and length are ~14 and 33 Å, respectively. Here, D is the initial CNT defect zone diameter, d_p-NT_ is the equilibrium distance between the DOX planar fragment and the CNT sidewall surface, d′_p-NT_ is the equilibrium distance between the same point of the DOX planar fragment and the opposite CNT sidewall surface, and d_N-NT_ is the equilibrium distance between the DOX-nitrogen atom and the CNT sidewall surface. All distances and diameter are expressed in Å.

Run	Type	PB	GB	D	d_p-NT_	d′_p-NT_	d _N-NT_
1	Z(18,0)0N-DoxIn.v1	−108.6	−112.3	14.82	3.49		3.35
2	Z(18,0)0N-Hk1-DoxOut	−39.8	−38.9	14.78	3.38		4.56
3	Z(18,0)0N-Hk1-DoxDIn.v1	−104.6	−108.0	14.78	3.40	3.71	3.07
4	Z(18,0)0N-Hk1-DoxDIn.v2	−93.1	−94.5	14.78	3.77		3.39
5	Z(18,0)0N-Hk1-DoxRIn.v1	−93.6	−95.6	14.78	3.82		3.20
6	Z(18,0)0N-Hk1-DoxRIn.v2	−85.5	−86.2	14.78	3.59		3.66
7	Z(18,0)4N-Hk1-DoxDIn.v1	−108.4	−111.1	14.78	3.79	3.77	3.08
8	Z(18,0)8N-Hk1-DoxDIn.v1	−106.8	−110.5	14.78	3.64		3.38
9	Z(18,0)0N-DoxIn.v1RESP	−78.8	−79.0	14.82	3.71		4.00
10	Z(18,0)0N-Hk1-DoxOutRESP	−39.7	−37.7	14.78	3.54		3.92
11	Z(18,0)0N-Hk1-DoxDIn.v1RESP	−102.3	−104.5	14.78	3.89	4.95	3.33
12	Z(18,0)4N-Hk1-DoxDIn.v1RESP	−102.3	−104.7	14.78	3.69	3.89	3.08
13	Z(18,0)8N-Hk1-DoxDIn.v1RESP	−102.1	−103.1	14.78	3.70	3.62	4.20
14	Z(18,0)0N-Hk2-DoxDIn.v1	−85.9	−85.9	15.41	3.59		4.77
15	Z(18,0)0N-Hk2-DoxDIn.v2	−99.7	−103.6	15.41	3.22	4.33	3.25
16	Z(18,0)0N-Hk2-DoxRIn.v1	−92.3	−92.4	15.41	3.96		3.35
17	Z(18,0)0N-Hk2-DoxRIn.v2	−94.4	−95.6	15.41	3.79		3.49
18	Z(18,0)4N-Hk2-DoxDIn.v1	−98.0	−98.4	15.41	3.39		3.43
19	Z(18,0)8N-Hk2-DoxDIn.v1	−86.9	−87.3	15.41	3.48		2.97
20	Z(18,0)0N-Hk2-DoxDIn.v1RESP	−78.7	−79.0	15.41	3.83		2.99
21	Z(18,0)4N-Hk2-DoxDIn.v1RESP	−82.3	−81.9	15.41	3.61		3.83
22	Z(18,0)8N-Hk2-DoxDIn.v1RESP	−81.6	−80.9	15.41	3.57		3.14
23	Z(18,0)0N-H-Hk2-DoxDIn.v1RESP	−63.0	−58.1	15.41	3.58		3.30
24	Z(18,0)0N-H-Hk2-DoxDIn.v2RESP	−58.8	−54.3	15.41	3.65		2.98
25	Z(18,0)4N-H-Hk2-DoxDIn.v1RESP	−55.9	−51.4	15.41	4.11		3.36
26	Z(18,0)4N-H-Hk2-DoxDIn.v2RESP	−61.1	−55.7	15.41	3.59		4.32
27	Z(18,0)8N-H-Hk2-DoxDIn.v1RESP	−58.2	−53.5	15.41	3.62		4.41
28	Z(18,0)8N-H-Hk2-DoxDIn.v2RESP	−58.3	−47.8	15.41	3.83		4.27

**Table 7:** Poisson-Boltzmann (PB) and Generalized Bond (GB) binding free energy values for encapsulation DOX-CNT complexes of *zigzag* (18,0) nanotubes with two haeckelite defects expressed in kcal/mol, at different simulation times expressed in ns. Nanotube diameter and length are ~14 and 33 Å, respectively. Here, d_p-NT_ is the equilibrium distance between the DOX planar fragment and the CNT sidewall surface, and d_N-NT_ is the equilibrium distance between the DOX-nitrogen atom and the CNT sidewall surface. All distances are expressed in Å.

Run	Simulation Time (ns)	PB	GB	d_p-NT_	d_N-NT_
Z(18,0)0N-Hk2-DoxRIn.v2					
1	2	−94.4	−95.6	3.79	3.49
2	5	−97.6	−98.9	3.89	3.94
3	30	−99.0	−100.6	3.74	3.47
4	50	−99.0	−100.6	3.58	3.83
5	100	−99.0	−100.6	3.56	3.38
Z(18,0)0N-Hk2-DoxDIn.v1					
6	2	−85.9	−85.9	3.59	4.77
7	5	−87.0	−87.0	3.86	3.20
8	10	−87.0	−87.0	3.38	3.90
9	15	−87.0	−87.0	3.99	3.20
